# Long-term outcomes of intervention between open repair and endovascular aortic repair for descending aortic pathologies: a propensity-matched analysis

**DOI:** 10.1186/s12893-020-00923-4

**Published:** 2020-11-03

**Authors:** Shin-Ah Son, Hanna Jung, Joon Yong Cho

**Affiliations:** 1 Trauma Center, Department of Thoracic and Cardiovascular Surgery, School of Medicine, Kyungpook National University, Kyungpook National University Hospital, Daegu, Republic of Korea; 2 Department of Thoracic and Cardiovascular Surgery, School of Medicine, Kyungpook National University, Kyungpook National University Hospital, 130 Dongdeok-ro, Jung-gu, Daegu, Republic of Korea

**Keywords:** Descending aorta, Endovascular repair, Graft replacement, Outcomes, Reintervention

## Abstract

**Background:**

The long-term complication rates of open repair and thoracic endovascular aortic repair (TEVAR) have not yet been determined. Therefore, this study aimed to compare the long-term outcomes and aortic reintervention rates between open repair and TEVAR in patients with descending thoracic aortic pathologies.

**Methods:**

Between January 2002 and December 2017, 230 patients with descending thoracic aortic pathologies underwent surgery. Of these, 136 patients were included in this retrospective study: 45 patients (10, 2, and 33 with dissection, penetrating atherosclerotic ulcer, and pseudoaneurysm, respectively) underwent open repair and 91 patients (27, 1, and 63 with dissection, penetrating atherosclerotic ulcer, and pseudoaneurysm, respectively) underwent TEVAR. The primary end points were in-hospital mortality, and short-term complications. The secondary end points were long-term mortality and reintervention rates. Based on the propensity score matching (PSM), 35 patients who underwent open repair were matched to 35 patients who underwent TEVAR (ratio = 1:1).

**Results:**

The mean follow-up period was 70.2 ± 51.9 months. Shorter intensive care unit and hospital stay were seen in the TEVAR group than in the open repair group before and after PSM (*p* < 0.001 and *p* < 0.001, respectively). However, in-hospital mortality, and spinal cord ischemia were not significantly different among the two groups (before PSM: *p* = 0.068 and *p* = 0.211, respectively; after PSM: *p* = 0.303 and *p* = 0.314, respectively). The cumulative all-cause death and aorta-related death showed no significant differences between the two groups (before PSM: *p* = 0.709 and *p* = 0.734, respectively; after PSM: *p* = 0.888 and *p* = 0.731, respectively). However, aortic reintervention rates were higher in the TEVAR group than in the open repair group before and after PSM (*p* = 0.006 and *p* = 0.013, respectively).

**Conclusion:**

The TEVAR group was superior in short-term recovery outcomes but had higher reintervention rates compared to the open repair group. However, there were no significant differences in long-term survival between the two groups.

## Background

The treatment of descending thoracic aortic pathologies is challenging and depends on its pathologies. Since the introduction of thoracic endovascular aortic repair (TEVAR), many reports have demonstrated that it is a safe and feasible alternative to the conventional open repair [1–3]. Typically, the success of this procedure is dictated by its favorable outcomes and ease of use [4]. Although the use of TEVAR has rapidly increased due to improved perioperative morbidity rates, significant postoperative complications associated with TEVAR contribute to its relatively poor results; these complications include, endoleak, stent-graft migration, retrograde type aortic dissection, new-onset dissection, and stent-graft infection [3, 5, 6]. In early clinical results with open repair versus TEVAR covered in previous reports [3, 6, 7], there are little long-term data comparing two procedures. In particular, the durability and long-term complication rates of open repair and TEVAR have not yet been determined. We hypothesized that the advantages of TEVAR, which included less invasive and ease of use, will result in improved long-term outcomes for patients.

The purpose of this study was to compare long-term outcomes and reintervention rates between open repair and TEVAR in patients with descending aortic pathologies. To neutralize the effects of confounding independent variables such as unbalanced numbers (45:91) and age discrepancy, a propensity matched subsample of patients was created for an adequately powered analysis.

## Methods

### Study population

**Fig. 1:**
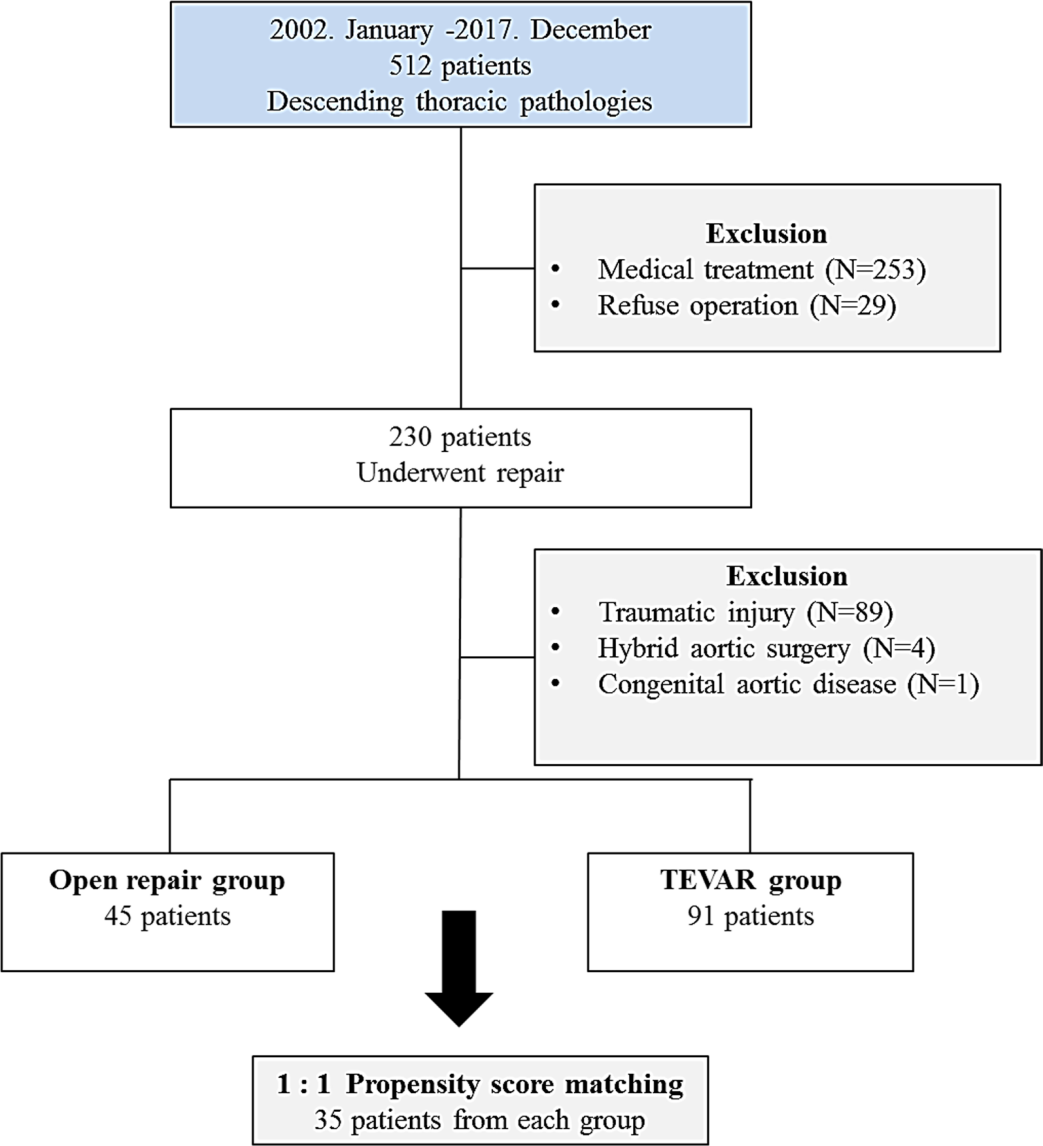
Study design flowchart

Between January 2002 and December 2017, 512 patients were diagnosed with descending thoracic aortic pathologies, comprising mainly dissection, pseudoaneurysm and penetrating atherosclerotic ulcer (PAU) at Kyungpook National University. Among them, 253 patients with uncomplicated descending aortic pathologies and who underwent medical therapy aimed at maintaining hemodynamic stability for promoting aortic stability were excluded; antihypertensive agents were administered to prevent aortic expansion. Furthermore, 29 additional patients who refused operation due to old age, financial problems, and poor general conditions were excluded as well. Out of the remaining 230 patients, 93 with traumatic aortic injury and hybrid aortic surgery who experienced cardiac arrest were excluded. Additional exclusion criteria were congenital aortic surgery including repair of coarctation of the aorta. The remaining 136 patients were included: Of these, 45 patients (10, 2, and 33 with dissection, PAU, and pseudoaneurysm, respectively) underwent open repair (the open repair group) and 91 patients (27, 1, and 63 with dissection, PAU, and pseudoaneurysm, respectively) underwent TEVAR (the TEVAR group). Telephonic clinical assessments and outpatient clinical records were reviewed for these patients. The study design flowchart is demonstrated in Fig. 1.

Acute dissection was defined when the occurrence developed within 14 days from the first symptom. Complicated aortic pathologies were defined as the presence of one or more of the following conditions: aortic maximum size > 5.5 cm; resistant hypertension despite adequate medical therapy; recurrent or refractory pain; impending rupture; rupture with end-organ malperfusion; and extension of dissection. Aortic reintervention was delineated as the need for any surgical or endovascular interventions following the initial procedure during follow-up. In the TEVAR group, an endoleak represents as radiological evidence of blood flow outside the stent -graft according to published guidelines [8].

### Operative strategies

Contrast-enhanced computed tomography (CT) was performed for the preoperative assessment of descending thoracic aortic pathologies. Surgery was performed for patients with complicated acute or chronic descending aortic pathologies. The treatment modality was decided collaboratively by the cardiologist and cardiac surgeons who were involved in the patients’ care; the decision was based on the patients’ co-morbidities, functional status, anatomical feature of the lesion, and the appropriate of vascular access [9].

Open aortic repair was performed via left thoracotomy or median sternotomy. All procedures were performed with sequential clamping to minimize ischemic times. In case of left atrial-left femoral artery partial bypass, blood was drained from the left atrium via the inferior pulmonary vein and returned through the femoral artery. In case of circulatory arrest, bypass was initiated via the femoral artery and vein or the ascending aorta and right atrium. After the distal aortic arch was cross-clamped, the aorta was opened and a proximal anastomosis was constructed. If there was severe calcification, and friable tissue of the aorta or proximal clamp technically impossible, the patient was put under total circulatory arrest. Moderate hypothermia to 24 °C was achieved by gradual cooling, and circulation was arrested [10].

Preoperative CT were reviewed for the preoperative assessment of access routes for the feasibility of TEVAR. The scans were also reviewed to investigate the bilateral vertebral artery for assessing the subclavian steal syndrome with a policy of selective subclavian artery revascularization. All TEVAR procedures were performed via the transfemoral approach under general anesthesia. Perioperative anticoagulation with heparin was prescribed at a dose of 3000–5000 units. For patients with a healthy native aorta, stent-graft oversizing was considered 5–10%, and excessive oversizing was considered over 20%. The proximal landing zones in the aortic arch were classified as 0 to 4 according to Ishimaru’s classification [11]. The proximal landing zone length was maintained at least 2 cm away from the lesion, and bypass was performed if necessary, through a preoperative assessment. TEVAR was performed using S&G SEAL Thoracic Stent-Grafts (S&G Biotech, Seongnam, Korea) and Valiant Thoracic Stent-Grafts (Medtronic Vascular, Santa Rosa, CA, USA).

When comparing continuous variables, the Student’s t-test and Wilcoxon test were used for parametric and nonparametric data, respectively, and are presented with the mean ± standard deviation (SD) or as median and interquartile range (IQR). Categorical variables were reported as absolute numbers or percentages and the Fisher’s exact test or Chi-square test was used for comparison. The Kaplan–Meier method was used to estimate survival. For statistical analyses, *p*-values < 0.05 were deemed significant. Univariate and multivariate logistic regression models were utilized to determine independent risk factors. Hazard ratios (HRs) are presented with 95% confidence intervals (CIs).

To reduce the effect of selection bias and potential confounding in this retrospective cohort study, estimated propensity scores were used to match two groups. This was computed for each patient using a logistic regression model including the following variables: age, proximal maximal aortic size, aortic pathology, and proximal aortic tear site. The propensity score model was well-calibrated (Hosmer–Lemeshow goodness‐of‐fit test; *p* = 0.784) with good discrimination (c‐statistic = 0.712). To neutralize the effects of confounding variables, 35 patients in the open repair group were matched to 35 patients who underwent TEVAR using propensity score matching (PSM). The data were analyzed using SAS/STAT software, v. 9.4 (SAS Institute Inc., NC, USA) and SPSS 25 (IBM, Armonk, NY, USA).

## Results

### Baseline characteristics of patients

Baseline characteristics of the patients who underwent open repair and TEVAR are shown in Table 1. The mean follow-up duration was 70.2 ± 51.9 months (range: 0.0–212.0 months). The median age of the patients differed significantly between the open repair and TEVAR groups, and were 56.0 years (range: 43.0–64.0 years) and 65.0 years (range: 57.0–72.0 years), respectively (p < 0.001). Moreover, the incidence of connective tissue diseases was significantly higher in the open repair group than in the TEVAR group (p < 0.001). After PSM, the baseline characteristics of the patients exhibited no significant differences between the two groups.

**Table 1:** Baseline characteristics of patients

Characteristics	Overall series			After matching		
	Open repair N = 45	TEVAR N = 91	*p*-value	Open repair N = 35	TEVAR N = 35	*p*-value
Age (years)	56.0 (43.0–64.0)	65.0 (57.0–72.0)	* < 0.001*	60.0 (49.0–69.0)	59.0 (51.0–68.0)	0.967
Men	31 (30.1)	72 (69.9)	0.279	25 (71.4)	30(85.7)	0.145
Height (cm)	167 ± 10.0	166 ± 9.0	0.623	164.8 ± 10.1	168.3 ± 7.6	0.112
Weight (kg)	66.1 ± 13.2	64.9 ± 11.0	0.584	65.7 ± 12.9	68.9 ± 12.4	0.283
Initial SBP (mmHg)	133.5 ± 34.2	141.3 ± 29.3	0.208	135.7 ± 37.3	145.5 ± 27.1	0.215
Hypertension	30 (66.7)	66 (72.5)	0.480	26 (74.3)	25 (71.4)	0.788
Diabetes	5 (11.1)	12 (13.2)	0.731	5 (14.3)	1 (2.9)	0.088
Current smoking	20 (44.4)	36 (39.6)	0.586	16 (45.7)	16 (45.7)	1.000
Obesity	2 (4.4)	3 (3.3)	0.738	2 (5.7)	2 (5.7)	1.000
CAOD	4 (8.9)	10 (11.0)	0.705	3 (8.6)	6 (17.1)	0.284
PAOD	4 (18.2)	18 (81.8)	0.105	4 (11.4)	5 (14.3)	0.721
COPD	2 (4.4)	4 (4.4)	0.990	2 (5.7)	2 (5.7)	1.000
Cerebrovascular accident	1 (2.2)	11 (12.1)	0.056	1 (2.9)	4 (11.4)	0.164
Acute kidney injury	2 (4.4)	5 (5.5)	0.794	2 (5.7)	1 (2.9)	0.555
Chronic renal failure	4 (8.9)	7 (7.7)	0.810	2 (5.7)	3 (8.6)	0.643
Previous cardiac operation	10 (22.2)	18 (19.8)	0.740	3 (8.6)	6 (17.1)	0.284
Connective tissue disease	8 (17.8)	1 (1.1)	* < 0.001*	2 (5.7)	0 (0.0)	0.151
Preoperative status						
Shock	7 (15.6)	5 (5.5)	0.052	5 (14.3)	3 (8.6)	0.654
Hemoptysis	6 (13.3)	11 (12.1)	0.836	4 (11.4)	4 (11.4)	1.000
Persistent pain	37 (82.2)	73 (80.2)	0.780	30 (85.7)	27 (77.1)	0.356
Neurologic deficit	3 (6.7)	3 (3.3)	0.368	2 (5.7)	0 (0.0)	0.151

Values are presented as the median (interquartile range), mean ± standard deviation or number of patients (%)

*TEVAR* thoracic endovascular aortic repair, *SBP* systemic blood pressure, *CAOD* coronary artery occlusive disease, *PAOD* peripheral artery occlusive disease, *COPD* chronic obstructive pulmonary disease

Descending thoracic aortic pathologies details of the patients are shown in Table 2. No variables differed significantly between the two groups, both before and after PSM.

**Table 2:** Descending thoracic aortic diseases details of patients

Characteristics	Overall series			After matching		
	Open repair N = 45	TEVAR N = 91	*p*-value	Open repair N = 35	TEVAR N = 35	*p*-value
Acute	15 (33.3)	20 (22.0)	0.154	13 (37.1)	7 (20.0)	0.112
Rupture	19 (42.2)	21 (23.1)	*0.021*	16 (45.7)	9 (25.7)	0.081
Preoperative diagnosis						
Dissection	10 (22.2)	27 (29.7)	0.358	8 (22.9)	10 (28.6)	0.584
PAU	2 (4.4)	1 (1.1)	0.211	2 (5.7)	0 (0.0)	0.151
Pseudoaneurysm	33 (73.3)	63 (69.2)	0.621	25 (71.4)	25 (71.4)	1.000
Arch involvement	12 (26.7)	14 (15.4)	0.115	9 (25.7)	4 (11.4)	0.124
Maximal aortic size (mm)	54.9 ± 4.7	51.5 ± 4.5	0.471	52.8 ± 4.8	51.8 ± 3.7	0.985
Aortic tear site						
Arch	5 (11.1)	4 (4.4)	0.138	4 (11.4)	1 (2.9)	0.164
Isthmus	8 (17.8)	34 (37.4)	*0.020*	6 (17.1)	12 (34.3)	0.101
Descending	32 (71.1)	53 (58.2)	0.145	25 (71.4)	22 (62.9)	0.445
Malperfusion	5 (11.1)	3 (3.3)	0.068	3 (8.6)	0 (0.0)	0.077
CSF drainage	14 (31.1)	17 (18.7)	0.104	8 (22.9)	7 (20.0)	0.771
Emergency	17 (37.8)	28 (30.8)	0.414	16 (45.7)	9 (25.7)	0.081

Values are presented as mean ± standard deviation or number of patients (%)

*TEVAR* thoracic endovascular aortic repair, *PAU* penetrating atherosclerotic ulcer, *CSF* cerebrospinal fluid

Operative details are shown in Table 3. In the open repair group, circulatory arrest perfusion was performed most frequently (60.0%), and thoracotomy was the most common approach (60.0%). In the TEVAR group, zone 3 TEVAR was performed most frequently (40.7%).

**Table 3:** Operative details

Open repair	(N = 45)
Perfusion method	
Left atrium- Femoral artery partial bypass	15 (33.3)
Partial Shunt^a^	3 (6.67)
Circulatory arrest	27 (60.0)
Femoral artery-Femoral vein	9 (20.0)
Aorta-Right atrium	18 (40.0)
Operative Approach	
Median sternotomy	10 (22.2)
Median sternotomy + Thoracotomy	8 (17.7)
Thoracotomy	27 (60.0)
Cardiopulmonary bypass time (min)	191.1 ± 104.3
Cross clamp time (min)	60.5 ± 49.9
Circulatory arrest time (min)	25.5 ± 31.4
TEVAR	(N = 91)
Number of devices	1.2 ± 0.4
Proximal stent size (mm)	35.1 ± 4.7
Stent Length (mm)	140.2 ± 28.2
Zone 0	6 (6.6) Total debranching bypass with TEVAR
Zone 1	1 (1.1) BCA to LCCA and LCCA to LSCA bypass
Zone 2	25 (27.5) LCCA to LSCA bypass in 5 patients
Zone 3	37 (40.7)
Zone 4	22 (24.2)

Values are presented as mean ± standard deviation or number of patients (%)

*TEVAR* thoracic endovascular aortic repair, *BCA* brachiocephalic artery, *LCCA* left common carotid artery, *LSCA* left subclavian artery

^a^Graft placed proximal and distal to the injury site

### Postoperative outcomes and complications

Postoperative outcomes and complications are shown in Table 4. The patients in the open repair group significantly required more operative time, needed longer ventilator care, stayed longer in the intensive care unit, and had longer periods of hospitalization than those in the TEVAR group (all p < 0.05, respectively), before and after PSM, respectively.

**Table 4:** Postoperative outcomes and complications

Characteristics	Overall series			After matching		
	Open repair N = 45	TEVAR N = 91	*p*-value	Open repair N = 35	TEVAR N = 35	*p*-value
Outcomes						
Operative time	420.6 ± 182.9	149.3 ± 92.9	< *0.001*	386.7 ± 124.5	150.9 ± 88.7	< *0.001*
Postop Hospital stay (day)	41.5 ± 42.7	18.3 ± 16.0	< *0.001*	34.2 ± 26.5	15.1 ± 8.6	< *0.001*
Total ICU stay (day)	19.6 ± 31.2	5.7 ± 14.0	< *0.001*	17.7 ± 24.6	3.7 ± 5.4	*0.002*
Total ventilator care (min)	145.7 ± 334.1	33.6 ± 158.2	*0.009*	111.9 ± 225.4	11.9 ± 48.6	*0.008*
Reintubation	7 (15.6)	4 (4.4)	*0.025*	5 (14.3)	1 (2.9)	0.088
Complications						
Acute kidney injury	19 (42.2)	18 (19.8)	*0.006*	15 (42.9)	6 (17.1)	*0.019*
Dialysis	6 (13.3)	6 (6.6)	0.192	3 (8.6)	1 (2.9)	0.303
Hematologic complications	1 (2.2)	2 (2.2)	0.993	1 (2.9)	0 (0.0)	0.314
Bleeding	9 (20.0)	3 (3.3)	*0.001*	6 (17.1)	2 (5.7)	0.133
Spinal cord injury	2 (4.4)	1 (1.1)	0.211	1 (2.9)	0 (0.0)	0.314
Stroke	3 (6.7)	1 (1.1)	0.071	2 (5.7)	0 (0.0)	0.151
Pulmonary complications	9 (20.0)	7 (7.7)	*0.036*	7 (20.0)	2 (5.7)	0.074
Wound complications	12 (26.7)	6 (6.6)	*0.001*	9 (25.7)	3 (8.6)	0.057
In-hospital mortality	5 (11.1)	3 (3.3)	0.068	3 (8.6)	1 (2.9)	0.303
30-day mortality	5 (11.1)	4 (4.4)	0.138	3 (8.6)	2 (5.7)	0.643

Values are presented as mean ± standard deviation or number of patients (%)

*TEVAR* thoracic endovascular aortic repair, *Postop* postoperative, *ICU* intensive care unit

Postoperative acute kidney injury (AKI) was higher in the open repair group before and after PSM (*p* = 0.006 and *p* = 0.019, respectively); however, there was no difference between the two groups in the AKI requiring dialysis before and after PSM (*p* = 0.192 and *p* = 0.303, respectively). AKI progressed to chronic renal failure (CRF) in only two patients in the TEVAR group. Bleeding and wound complications including wound dehiscence and infection, were frequently observed in the open repair group before PSM (*p* = 0.001 and *p* = 0.001, respectively); however, there were no statistical difference between the two groups after PSM (*p* = 0.133 and *p* = 0.057, respectively). In addition, there was no statistical difference in occurrence of spinal cord ischemia (SCI) between the two groups before and after PSM (*p* = 0.211 and *p* = 0.314, respectively).

There were no statistical difference in-hospital mortality and 30-day mortality between the two groups before PSM (*p* = 0.068 and *p* = 0.138, respectively). Moreover, same as before PSM, in-hospital mortality and 30-day mortality showed no statistical difference between the two groups (*p* = 0.303 and *p* = 0.643, respectively).

In-hospital mortality was observed in five patients in the open repair group (11.1%), of whom four and one died of acute aorta-related complications and hospital-acquired pneumonia, respectively. In the TEVAR group, among three patients (3.3%), two patients died of delayed rupture after stent insertion and one patient died of aspiration pneumonia.

### Reintervention

Reintervention data associated with specific treatments are depicted in Table 5. In the open repair group, five patients (11.1%) required reinterventions; and four patients experienced new onset aortic expansion. Among these patients, secondary open repair surgery was performed in three patients, and TEVAR was performed in one patient. In the TEVAR group, 22 patients (48.9%) required reintervention; twelve patients had endoleaks and four patient developed fistula, such as aortobronchial fistula or aortoesophageal fistula.

**Table 5:** Aortic reintervention details

Cause of Reintervention	Open repair (N = 5, 11.1%)	TEVAR (N = 22, 48.9%)
New onset dissection/expansion	Aorta replacement:3 TEVAR:1	Aorta replacement:2 TEVAR:1
Fistula formation	Aorta replacement and lobectomy:1	Aorta replacement and lobectomy:4 Aorta replacement and esophagotomy:1 TEVAR:1
Endoleak		TEVAR:12 LSCA plug obliteration:1
Infection		Aorta replacement:1

*TEVAR* thoracic endovascular aortic repair, *LSCA* left subclavian artery

**Fig. 2:**
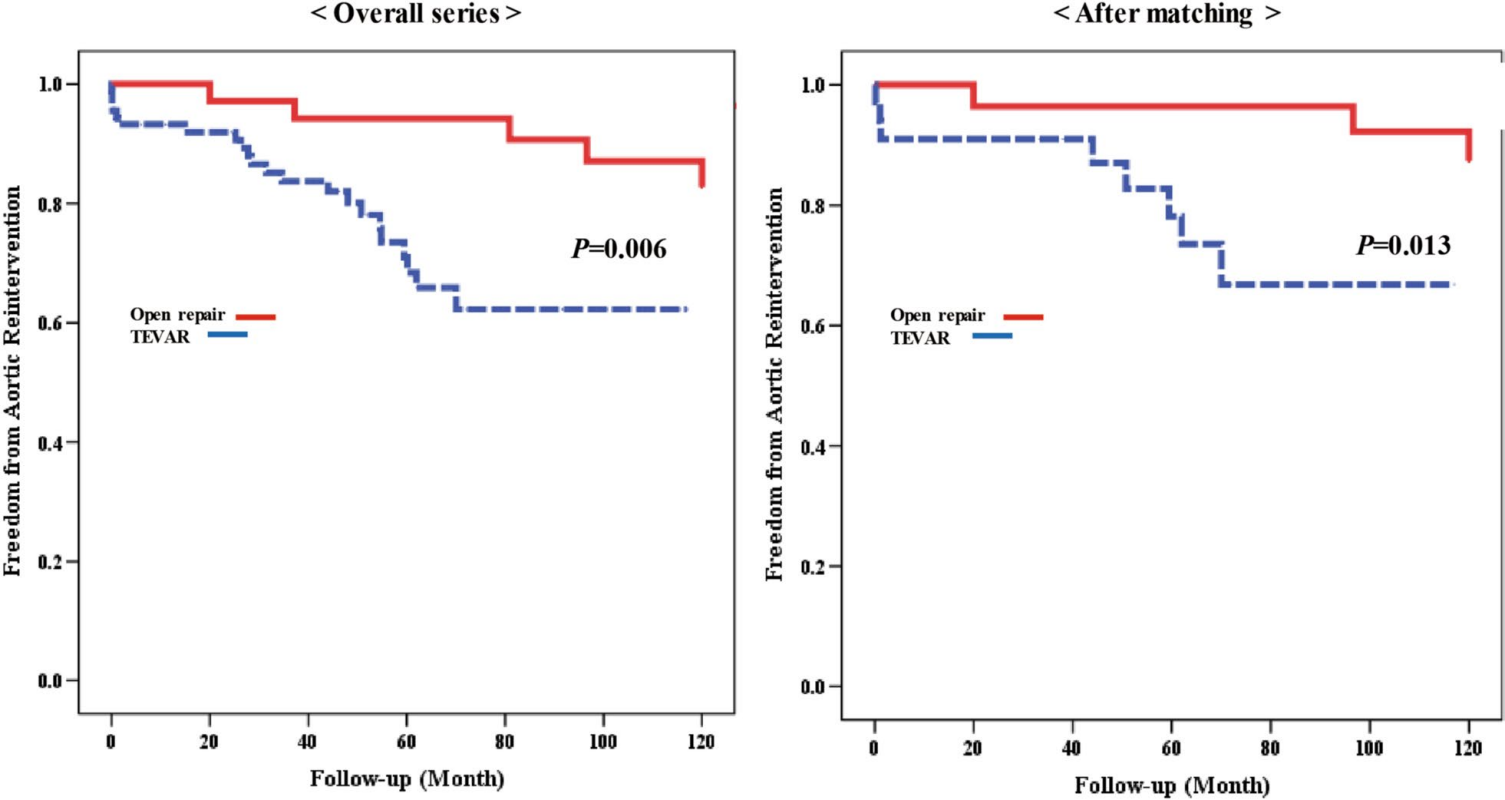
Freedom from aortic reintervention before propensity matching and after propensity matching

In the overall series, freedom from aortic reintervention at 1, 5, and 10 years in the open repair group was 97.1% ± 0.1%, 94.2% ± 0.1%, and 87.1% ± 0.0%, respectively. The corresponding rates in the TEVAR group were 93.2% ± 0.1%, 68.4% ± 0.1%, and 62.2% ± 0.0%, respectively. After PSM, the rate of freedom from aortic reintervention at 1, 5, and 10 years in the open group was 100% ± 0.0%, 96.4% ± 0.1%, and 92.2% ± 0.1%, respectively. In the TEVAR group, the corresponding values were 91.0% ± 0.1%, 78.1% ± 0.1%, and 66.8% ± 0.1%, respectively. Freedom from aortic reintervention (Fig. 2) was lower in the TEVAR group than in the open repair group before and after PSM (*p* = 0.006 and *p* = 0.013, respectively).

A multivariable Cox proportional hazard model is shown in Table 6.

**Table 6:** Cox proportional hazard regression analysis for reintervention

Variable	Overall series				After matching			
	Univariate		Multivariate		Univariate		Multivariate	
	HR (95% CI)	*p*-value	HR (95% CI)	*p*-value	HR (95% CI)	*p*-value	HR (95% CI)	*p*-value
Male	2.0 (0.6–5.8)	0.191			0.2 (0.0–2.2)	0.230		
Hemoptysis	2.8 (1.3–6.9)	0.026	3.8 (1.2–11.3)	*0.016*	8.5 (2.4–29.3)	0.001	6.8 (1.3–34.7)	*0.021*
Hoarseness	2.2 (0.8–9.0)	0.102			1.6 (0.2–13.2)	0.619		
Hypertension	0.2 (0.5–2.9)	0.595			4.4 (0.5–34.9)	0.151	7.9 (0.8–73.1)	*0.048*
Diabetes	2.0 (0.7–5.4)	0.148			1.3 (0.2–10.7)	0.766		
CAOD	2.5 (0.9–6.7)	0.059			2.2 (0.5–10.6)	0.210		
PAOD	1.7 (0.7–4.4)	0.210			1.9 (0.4–8.9)	0.400		
COPD	3.4 (1.0–11.4)	0.046			4.4 (0.9–21.2)	0.063		
Cerebrovascular accident	2.2 (0.7–6.4)	0.144			1.7 (0.2–14.2)	0.579		
Acute kidney injury	1.1 (0.1–7.4)	0.994			5.1 (0.6–41.2)	0.124		
Previous cardiac operation	1.1 (0.6–3.4)	0.388			3.2 (0.8–12.4)	0.082	6.81 (1.3–34.9)	*0.021*

*HR* hazard ratio, *CI* confidence interval, *CAOD* coronary artery occlusive disease, *PAOD* peripheral artery occlusive disease, *COPD* chronic obstructive pulmonary disease

### Change in the aorta size after the repair

**Fig. 3:**
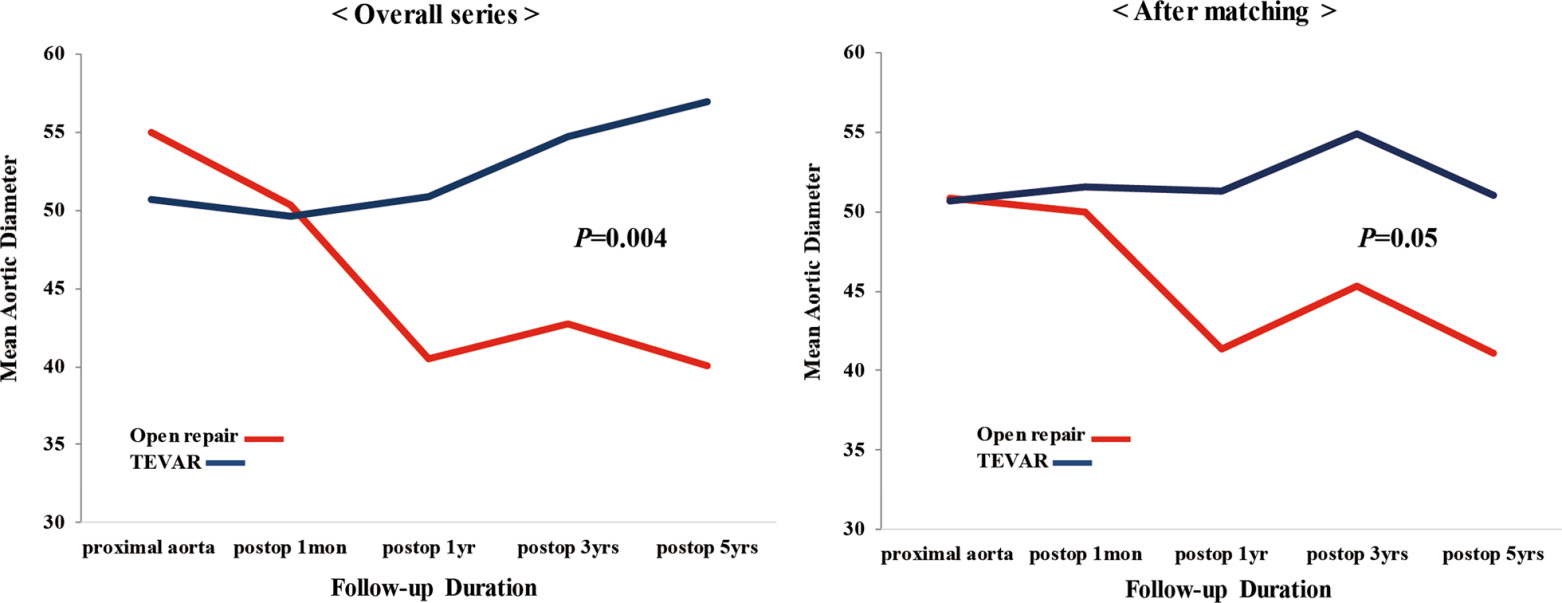
Changes in aortic diameters over time before propensity matching and after propensity matching

Figure 3 shows the changes in the aorta size after the repair. During the following 5 years, the maximal aortic diameter was more reduced in the open repair group as compared to that in the TEVAR group before and after PSM (*p* = 0.004 and *p* = 0.05, respectively). The maximal aortic diameter decreased from 55.4 ± 18.2 mm to 40.07 ± 11.0 mm in the open repair group as we expected. Meanwhile the maximal aortic diameter increased from 52.0 ± 14.7 mm to 56.8 ± 19.1 mm in the TEVAR group.

### Survival

In the open repair group, the overall mortality was 42.2% (19/45), and the aorta-related mortality was 17.8% (8/45) during follow-up. Aorta-unrelated death secondary to cancer occurred in three patients, while three patients died of pneumonia. Late deaths secondary to unknown causes occurred in four patients. In the TEVAR group, the overall mortality was 35.2% (32/91), and the aorta-related mortality was 12.1% (11/91) during follow-up. Aorta-unrelated death secondary to cancer occurred in three patients, while two patients died of pneumonia. Late deaths secondary to unknown causes occurred in 10 patients.

**Fig. 4:**
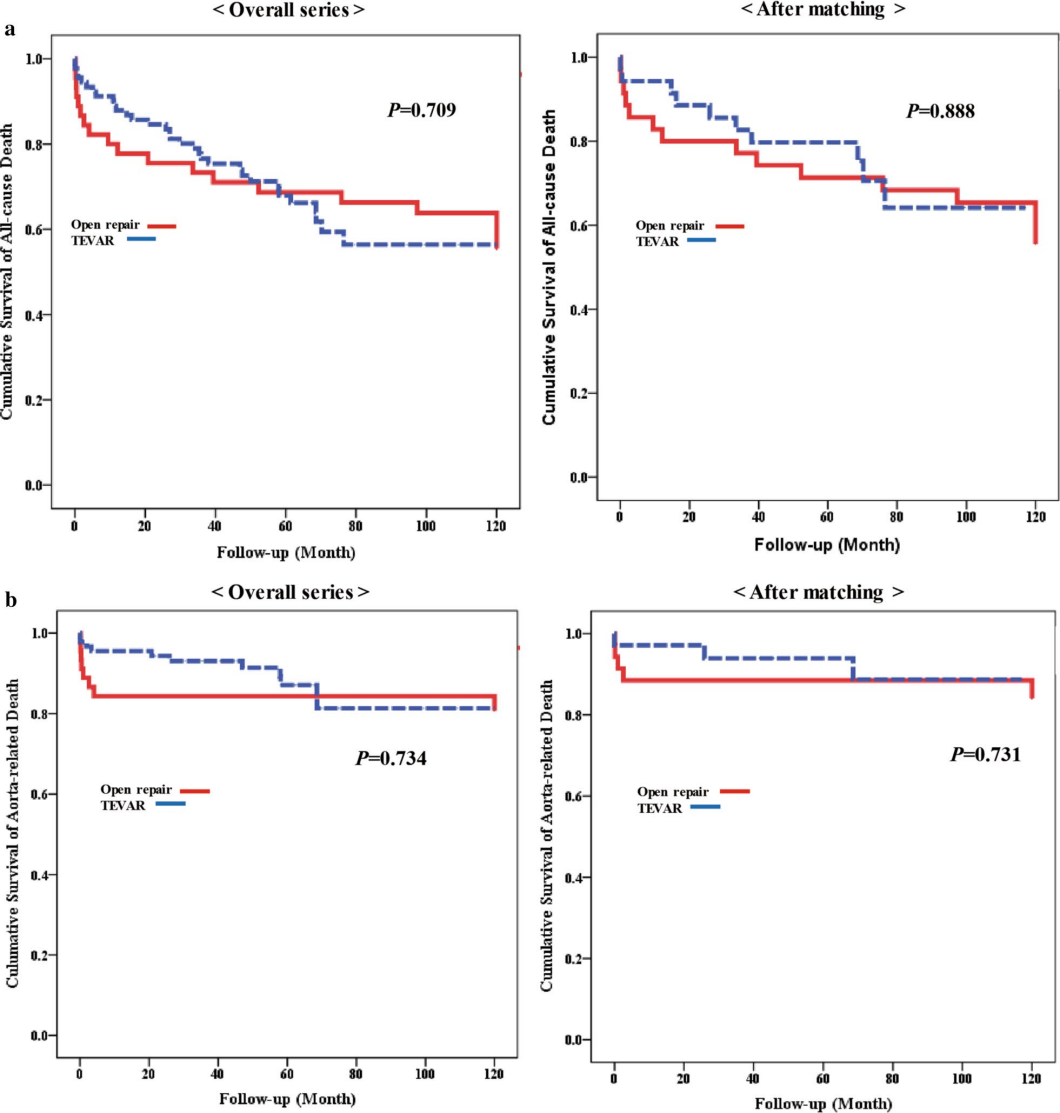
Cumulative survival of all-cause death and aorta-related death

The cumulative survival of all-cause death did not differ significantly between the two groups, neither before nor after PSM. Additionally, the cumulative survival rates from aorta-related deaths were also not significantly different between the two groups, before and after PSM (Fig. 4a, b).

A multivariable Cox proportional hazard model identified that age > 80 years, systolic blood pressure < 90 mmHg, diabetes, preoperative chronic renal failure, and aortic arch involvement were the predictive factors in the in overall series; after PSM analysis, age > 80 years, and aortic arch involvement (p = 0.024 and p = 0.048, respectively) were the independent predictors of aorta-related mortality (Table 7).

**Table 7:** Cox proportional hazard regression analysis for aorta-related mortality

Variable	Overall series				After matching			
	Univariate		Multivariate		Univariate		Multivariate	
	HR (95% CI)	*p*-value	HR (95% CI)	*p*-value	HR (95% CI)	*p*-value	HR (95% CI)	*p*-value
Age > 80 years	5.9 (1.3–25.9)	0.018	16.7 (2.7–104.3)	*0.002*	9.9 (1.2–83.4)	0.031	24.0 (1.5–378.1)	*0.024*
SBP < 90 mmHg	2.9 (0.9–8.9)	0.058	6.0 (1.6–22.4)	*0.008*	2.6 (0.5–13.3)	0.241		
Hemoptysis	2.5 (0.9–7.0)	0.077			5.2 (1.2–22.0)	0.023		
Dissection	0.7 (0.3–1.4)	0.356			0.4 (0.0–3.3)	0.392		
Pseudoaneurysm	1.4 (0.7–2.6)	0.333			2.8 (0.3–23.2)	0.329		
Arch involvement	3.5 (1.4–8.7)	0.007	5.4 (1.8–16.1)	*0.002*	5.1 (1.3–20.9)	0.022	14.5 (1.0–211.5)	*0.048*
Maximal aortic size > 50 mm	3.1 (0.9–10.8)	0.068			3.8 (0.5–31.9)	0.200		
Malperfusion	2.3 (0.5–10.1)	0.210			5.2 (0.6–43.9)	0.128		
Chronic renal failure	7.0 (2.4–20.3)	0.000	5.7 (1.2–26.8)	*0.027*	8.4 (1.5–46.7)	0.015		
eGFR	1.2 (1.1–1.3)	0.001			1.1 (0.9–1.5)	0.211		
Diabetes	2.8 (1.0–7.8)	0.047	3.1 (0.9–10.7)	*0.049*	4.2 (0.8–20.9)	0.080		
COPD	2.7 (0.6–11.9)	0.178			0.1 (0.0–34.8)	0.654		

*HR* hazard ratio, *CI* confidence interval, *SBP* systolic blood pressure, *eGFR* estimated glomerular filtration rate, *COPD* chronic obstructive pulmonary disease

## Discussion

The prevalence of descending thoracic aortic pathologies, comprising mainly of aneurysm, dissection, and PAU, which eventually rupture if not recognized and treated appropriately, are increasing among our population [12]. Since, the report on the first successful open repair of a descending thoracic aortic aneurysm with a prosthetic graft in 1953 by De Bakey and Cooley [13], an open surgical repair for treating descending thoracic aortic disease has been the gold standard for 50 years [13–16]. Despite remarkably improved operative techniques and maximized organ protection, open repair of the descending thoracic aorta is still associated with high complications, including intraoperative and postoperative death, hemorrhage, stroke, and paraplegia [9, 17].

Dake et al. [14] proposed an alternate method of TEVAR which sought to provide better clinical outcomes in patients who were deemed to be at high risk for open repair or were typically considered nonsurgical candidates. Therefore, TEVAR has shown significantly improved early quality of life versus open repair and a general trend toward better short-term perioperative survival and freedom from major complications [1, 3, 4, 18]. However, TEVAR has anatomic restrictions such as severe thoracic aortic tortuosity, short landing and sealing zones, and extensive mural thrombus. These are the limiting factors, although a seemingly infinite variety of debranching and bypass procedures can be applied to extend either the proximal or distal sealing zones [6, 19]. Furthermore, significant complications related to stent-grafts were always implied [3, 5, 17].

Patients who underwent TEVAR have a tendency to have a worse prognosis and older age, with multiple comorbidities, than patients who underwent open repair. Due to the relative lack of data supporting the long-term reliability of TEVAR, open repair procedure has been preferentially offered to younger patients [4, 12]. Therefore, in this study, to neutralize the effects of age difference that could potentially unmask a mortality benefit, PSM was performed between the two groups to perform an adequately powered analysis.

In the present study, the operative time, postoperative length of stay, and procedure-related complications showed better results with TEVAR before and after PSM. Not surprisingly, TEVAR was considering as the procedure involved no aortic cross-clamping, ischemic time, or thoracotomy [4]. In open repair cases, some disadvantages of deep hypothermia, including coagulopathy which caused difficulty in controlling bleeding, retraction injury to a heparinized lung, cold injury to the lung, and a profound inflammatory response from the bypass circuit [20]. Regarding the in-hospital mortality in the open repair group, in the present study, one patient died of pneumonia. AKI is another important complication and regarded as a marker of increased early or late morbidity and mortality after open repair or TEVAR [21]. Patients who underwent TEVAR were older and tended to receive larger amounts of contrast agent, which was not safe considering the risk of AKI. In the present study, postoperative AKI was higher in the open repair group (42.2%), and dialysis was performed in 13.3% of the patients in this group. Eighteen patients who underwent TEVAR (19.8%) had an AKI, six patients in the TEVAR group experienced AKI requiring dialysis. Before and after PSM, AKI was higher in the open repair group; however, there was no statistical difference in the requirement of dialysis between the two groups.

Although a short-term hospital outcome is more favorable for TEVAR, aorta-related complications are more frequent for TEVAR. Five patients (11.1%) in the open repair group underwent reintervention, and the most common cause of reintervention was new-onset aortic dissection or expansion. In the case of TEVAR, the most common cause of reintervention was endoleaks. Twenty-two (48.9%) patients who underwent reintervention showed no in-hospital mortality in the TEVAR; however, seven patients showed late mortality, one patient died of aortobronchial fistula and one patient died of sepsis due to stent-graft infection. Ascending aortic replacement was performed in one patient with retrograde aortic dissection, four patients with aortabronchial fistula underwent aorta replacement and lobectomy, and one patient with aortoesophageal fistula underwent aorta replacement and esophagotomy, and showed late mortality. Additionally, eight patients who were initially offered TEVAR, later crossed over to open repair due to difficult anatomy or other reasons.

In the open repair cases, high-volume centers reported mortality and neurological morbidity rates ranging from 5.4% to 7.2% for mortality, 2.1% to 6.2% for permanent stroke, 5.7% for permanent paraparesis, and 0.8% to 2.3% for permanent paralysis [22, 23]. In the TEVAR cases, the perioperative results for the three stent-graft trials showed 1.9 to 2.1% for mortality, 2.4 to 4% for stroke, 4.4 to 7.2% for permanent paraparesis, and 1.3 to 3% for permanent paralysis [1, 2, 24]. In the present study, the open repair group showed 63.8% of overall 10-year survival rate, 84.3% of aorta-related 10-year survival rate, 6.7% of stroke, and 4.4% of SCI (after PSM, 65.4%, 88.5%, 5.7%, and 2.9%, respectively). In TEVAR, overall 10-year survival rate was 56.5%, aorta-related 10-year survival rate 81.3%, 1.1% stroke, and 1.1% SCI (after PSM, 64.2%, 88.7%, 0.0%, 0.0%, respectively). Although mortality was higher than previous large-scale studies, it was difficult to compare our results because previous reports did not have long-term follow-up data.

The existing literature on TEVAR versus open surgical repair suggests that the short-term benefit of TEVAR is lost as early as within 1–2 years from the surgery [3–7]. For mid-term outcomes, analysis of the Medicare database from 2004 to 2007 showed that TEVAR offered a significant perioperative survival advantage when compared to open repair, regardless of the indication for repair. However, the 5-year survival in the Medicare population was similar between the two cohorts [25]. In another study, Goodney et al. [26] reported patients with intact thoracic aortic aneurysm who underwent TEVAR had a significantly worse survival at 1 year than patients who underwent open repair (87% for open repair and 82% for TEVAR; *p* = 0.001) and 5 years (72% for open repair and 62% for TEVAR; *p* = 0.001) than patients who underwent open repair.

Chiu et al. [27] reported that open surgical repairs of descending thoracic aortic aneurysms between 1999 and 2010 were associated with increased odds of early postoperative mortality, but reduced late hazard of death. Despite the improved durability of open surgical repair, the initial mortality advantage of TEVAR over open surgical repair persisted for until nine years post-operatively, resulting in a significant survival benefit associated with TEVAR. They proposed TEVAR ought to be considered as first-line therapy among Medicare beneficiaries, and open surgical repair be restricted to high-volume centers and patients with a low risk of perioperative mortality. However, Desai et al. [13] reviewed their series of patients who had descending thoracic aortic aneurysms repaired by either TEVAR or open repair. They concluded that 105 patients in the TEVAR group clearly had undergone more reinterventions than 45 patients who underwent open repair. The TEVAR cohort had a trend towards a decreased 30-day mortality as well as decreased rates of neurologic complications; however, none of these findings reached statistical significance. During a long-term follow-up from 1995 to 2007, the choice between TEVAR and open approach did not influence survival (*p* = 0.5).

In our study, TEVAR was more prevalent in terms of postoperative short-term outcomes. However, we could not conclude that TEVAR was superior to open repair in terms of SCI, 30-day mortality, and in-hospital mortality. Furthermore, there was no significant difference in the long-term follow-up survival between the two approaches. We hypothesized that these advantages of TEVAR, i.e., less invasiveness and ease of use, will provide improved long-term results for the patients.

Similar to the previous study, our findings indicated that TEVAR was better than open repair in terms of short-term outcomes; however, the long-term results were similar in both groups. Considering the fact that the previous studies had data older than in this study and there were discrepancies in the age or number of patients between the two groups, we believe that this study, which used PSM to neutralize the effects of confounding independent variables, presents more accurate data on the long-term results of the two groups.

In case of reintervention rates, there were significantly reinterventions in the TEVAR group than in the open repair group. Moreover, although long-term survival of the two groups did not differ significantly, more reinterventions occurred in the TEVAR group; the costs of additional graft modules to treat endoleaks and follow-up CT scans increase hospital cost, attributing to the disadvantage of TEVAR. Furthermore, while TEVAR had lesser procedure-related complications than open repair; patients had more adverse events, such as re-dissection, fistula formation and stent-graft infection. This should be considered in the choice of approach.

Some authors have proposed that TEVAR does not change the natural history of the disease, and although less invasive, may be inferior to open therapies [27]. In our present study, the maximal aortic size decreased more in the open repair group than in the TEVAR group, but not dramatically. This supports the report that it does not alter natural history of aortic pathologies, and, emphasizes the importance of long -term follow up. For this reason, in patients requiring TEVAR, the establishment of a precise TEVAR indication will reduce the requirement for further reintervention; better results are expected with improvements in debranching skills and stent- graft development.

Our study has several limitations. First, it was a single-center retrospective study that included a small number of patients, with a possible selection bias that might have affected our results. Second, the difference of follow-up duration and frequency can affect the survival rate of both groups. We performed PSM attempts to reduce the bias due to confounding variables. However, since TEVAR was introduced in 2007, it has a relatively short follow-up duration, whereas more frequent follow-up to monitor stent- grafts is expected to affect the results. Finally, the functional status of patient information influenced treatment strategy; there was no data interpretation, and studies on cost analysis, which is an increasingly important consideration for treatment strategy, have not been conducted. The results, which include all of these, may influence the final assessment of quality of life and longer life expectancy.

## Conclusions

In conclusion, our study showed that a long-term comparison between open repair and TEVAR demonstrated similar results in patients with descending aortic pathologies. However, patients who underwent TEVAR showed superior short-term recovery outcomes and a higher reintervention rate than the open repair group. Larger multicenter population studies that consider quality of life could support our results.

## Data Availability

The datasets used and/or analyzed during the current study are available from the corresponding author on reasonable request. The datasets used and/or analyzed during the current study are available from the Department of Thoracic and Cardiovascular Surgery, Kyungpook National University, Kyungpook National University Hospital, on reasonable request.

## Abbreviations

TEVAR: Thoracic endovascular aortic repair
PSM: Propensity score matching
PAU: Penetrating atherosclerotic ulcer
CT: Computed tomography
SD: Standard deviation
IQR: Interquartile range
HRs: Hazard ratios
CIs: Confidence intervals
AKI: Acute kidney injury
CRF: Chronic renal failure
SCI: Spinal cord ischemia
